# Coexistence of carbapenem resistance and hypervirulence in clinical *Klebsiella pneumoniae* isolates: a molecular and phenotypic analysis

**DOI:** 10.1186/s12879-025-11585-z

**Published:** 2025-09-16

**Authors:** Hadir Karam Ahmed, Fatma Molham, Ahmed O. El-Gendy, Mohamed Abd El-Gawad El-Sayed Ahmed

**Affiliations:** 1 Department of Microbiology and Immunology, Faculty of Pharmacy, Beni-Suef University, Beni-Suef, 62514 Egypt; 2 Department of Microbiology and Immunology, Faculty of Pharmaceutical Sciences and Drug Manufacturing, Misr University for Science and Technology, 6th of October City, Giza, Egypt

**Keywords:** *Klebsiella Pneumoniae*, Carbapenem resistant, Hypervirulent strains, Multidrug resistance, Virulence genes

## Abstract

**Background:**

Carbapenem-resistant hypervirulent *Klebsiella pneumoniae* (CRhvKp) is increasingly recognized as the predominant nosocomial pathogen in healthcare environments.

**Objective:**

This study aims to investigate the prevalence, antibiotic resistance patterns, and molecular characteristics of CRhvKp in Egypt.

**Methods:**

Out of 150 non-duplicate clinical isolates collected from a hospital in Egypt, 63 were identified as *K. pneumoniae* via biochemical tests and matrix-assisted laser desorption/ionization–time-of-flight mass spectrometry (MALDI-TOF MS). The CRKp isolates were identified using the VITEK^®^2 system and KB disc diffusion. Subsequently, to define CRhvKp using PCR, a specific conjugation of *rmp*A and/or *rmp*A2 with *iuc*A, *iro*B, or *peg*-344 was employed. Virulence and carbapenemase genes for CRKp isolates were screened via PCR, and the results of the carbapenemase genes were validated through Sanger sequencing.

**Results:**

Among the 63 *K. pneumoniae* isolates, 30 were identified as CRKp, and all exhibited multidrug resistance (MDR). CRKp was classified as 66.7% for CRhvKp and 33.3% for CR-non-hvKp. The presence of various virulence genes in CRhvKp and CR-non-hvKp samples was observed as follows: *fim*H (75% and 60%), *mrk*D (100% and 90%), *ent*B (80% and 90%), *irp*2 (85% and 90%), *wab*G (90% and 60%), and *uge* (40% and 60%). The existence of carbapenemase genes in CRhvKp and CR-non-hvKp samples was observed as follows: *bla*_NDM_ (41.3% and 25.0%), *bla*_KPC_ (10% and 5%), and *bla*_OXA−48_ (20.0% and 0.0%).

**Conclusion:**

Our study identified a significant percentage of MDR CRhvKp and CR-non-hvKp isolates harboring different carbapenemases, which sparked substantial concern. Herein, we highlight the concerning rapid increase of CRhvKp in Egypt. Hence, continued countrywide are surveillance is crucial to clarify the prevalence rate of these CRhvKp in Egypt.

**Supplementary Information:**

The online version contains supplementary material available at 10.1186/s12879-025-11585-z.

## Introduction

*Klebsiella pneumoniae* (*K. pneumoniae*) is a Gram-negative pathogen that often poses an opportunistic threat, commonly associated with various infections acquired in hospital settings [1]. In the last twenty years, there has been a global rise in *K. pneumoniae* incidence, showing resistance to last-resort antibiotics, including carbapenem antibiotics [2]. *K. pneumoniae* has two distinct evolutionary genetic lineages: hypervirulent *K. pneumoniae* (hvKp) and classical *K. pneumoniae* (cKp) [3]. The cKp lineages are identified as nosocomial pathogens in immunodeficient patients that can cause serious illnesses, including meningitis, bacteremia, urinary tract infections, and pneumonia. HvKp strains can induce metastatic and life-threatening diseases in immunocompetent individuals and healthy young adults. Liu et al. were the first to identify variations in hvKP in 1986 from Taiwan [4]. In addition to being a major contributor to pyogenic liver abscesses (PLA), some of the hvkp have also been linked to endophthalmitis and metastatic meningitis [5]. Hvkp lineages have seldom been identified to carry multidrug-resistant (MDR) genes until the last ten years, unlike classical *K. pneumoniae*, which is often MDR and even carbapenem resistant [6].

Several biomarkers have recently demonstrated diagnostic accuracies greater than 0.95 for detecting hvKp strains. These include the regulator of mucoid phenotype A (*rmp*A), the regulator of mucoid phenotype A2 (*rmp*A2), the putative metabolite transporter (*peg*−344), the siderophore aerobactin (*iuc*A), and salmochelin (*iro*B) [7].

There is a correlation between the hypermucoviscous phenotype and the *rmp*A and *rmp*A2 genes [8, 9]. *Peg*−344, *iro*B, and *iuc*A are located on virulence plasmids related to the hypervirulent phenotype of *K. pneumoniae* [10]. Li et al. suggest that employing a conjugation of *rmp*A and/or *rmp*A2 together with *peg*−344, *iuc*A, or *iro*B to identify hvKp would yield more reliable results [11].

Various studies have demonstrated that carbapenem-resistant *K. pneumoniae* (CRKp), especially the hypervirulent variant (CRhvkp), is correlated with heightened death and illness [12]. CRhvKp can rapidly disseminate in clinical environments, resulting in lethal epidemics, as demonstrated by numerous studies. This hypothesis has attracted global attention [8, 13, 14]. CRhvkp has the potential to become the next “superbug,” presenting a significant threat to global public health [15].

This study seeks to examine the prevalence, antibiotic resistance patterns, and molecular features of CRhvKp in Egypt and identify the genetic and phenotypic markers associated with virulence in CRhvKp, comparing them with carbapenem-resistant-non-hypervirulent *K. pneumoniae* (CR-non-hvKp) isolates to improve understanding of this emerging pathogen’s threat in healthcare settings.

## Materials and methods

### Clinical specimens

A total of 150 non-duplicate clinical isolates were obtained from several sources, including blood, pus, urine, sputum, drains, and wounds of hospitalized patients in Misr University for Science and Technology Teaching Hospital (Giza, Egypt), from January 2022 to August 2022, as part of a study on antimicrobial resistance monitoring. The identification of species was mainly done using biochemical tests and Gram stain [16], and it was confirmed using matrix-assisted laser desorption/ionization–time-of-flight mass spectrometry (MALDI-TOF MS) performed by the VITEK MS system (bioMérieux, France).

### Phenotypic identification of resistance and virulence indication

AST was conducted via the Kirby-Bauer disc diffusion method according to the Clinical and Laboratory Standards Institute guidelines [17] for the following 16 different antibiotics discs obtained from Oxoid, UK: amoxicillin-clavulanate (20/10 µg), aztreonam (30 µg), nitrofurantoin (30 µg), piperacillin/tazobactam (100/10 µg), ceftazidime (30 µg), imipenem (10 µg), meropenem (10 µg), neomycin (30 µg), tobramycin (10 µg), amikacin (30 µg), azithromycin (15 µg), gentamicin (10 µg), cefepime (30 µg), trimethoprim/sulfamethoxazole (1.25/23.75 µg), tetracycline (30 µg), and ciprofloxacin (5 µg). *E. coli* ATCC 25,922 was utilized as the standard control strain. Any isolates showing resistance in disc diffusion, including those suspected to be carbapenem-resistant, were further screened using the automated VITEK^®^2 AST-16 Gram-negative susceptibility card (bioMérieux, France) to confirm the resistance pattern. The MIC of colistin was performed using the broth microdilution method and interpreted via the EUCAST 2019 guidelines (ECOFFs; http://www.eucast.org/).

CRKp isolates were evaluated for carbapenemase production via modified carbapenem inactivation method (mCIM) in combination with the EDTA-modified carbapenem inactivation method (eCIM) according to CLSI’s (2023) recommendations. Since the mCIM is excellent at detecting carbapenemases, it is unable to differentiate between serine and metallo-β-lactamases (MBLs), so further modification of this assay with the incorporation of EDTA was performed according to CLSI’s (2023) recommendations [17].

The hypermucoviscosity among our isolates was assessed phenotypically via the string test, as previously explained [18]. Briefly, all isolates were inoculated on a blood agar plate and incubated at 37 °C overnight. A positive string test will generate a viscous string ≥ 5 mm in length, which can be stretched upwards by an inoculation loop.

### Molecular identification of resistance and virulence markers in CRKp isolates

Genomic DNA was extracted from all CRKp isolates via the boiling technique [19]. Five specific biomarker genes were utilized to identify CRhvKp, namely *iuc*A, *rmp*A, *rm*pA2, *peg*−344, *and iro*B(Russo et al., 2018). Eight distinct virulence genes were used to assess virulence in our isolates: fimbriae (*fim*H, *mrk*D), the iron siderophore enterobactin biosynthesis gene (*ent*B), yersiniabactin biosynthesis gene (*irp*2), lipopolysaccharide biosynthesis genes (*wab*G and *uge*), and for capsular serotypes (K1, K2*)* were also included [20]. The primers and the product size are listed in Table 1.

CRKp isolates were investigated for genes encoding carbapenemases via PCR, specifically: class A carbapenemase gene (*bla*_KPC_), class B carbapenemase genes (*bla*_IMP_, *bla*_VIM_, and *bla*_NDM_), and class D carbapenemase gene (*bla*_OXA−48_). In addition, the *mcr*−1 gene, associated with plasmid-mediated colistin resistance, was also screened. All these genes were examined utilizing the primers listed in Table 1. Three different isolates, each positive for one of the carbapenemase genes (*bla*_KPC_, *bla*_NDM_, or *bla*_OXA−48_), were purified using the DNA Clean & Concentrator-25 kit (Zymo Research, California, USA) and subsequently dispatched to GATC Biotech for Sanger sequencing to verify the PCR results. We compared the sequences to those previously present in the GenBank database (http://blast.ncbi.nlm.nih.gov/Blast.cgi).

**Table 1:** List of primers applied in this study

Target gene	Sequences (5′–3′)	Annealing (◦C)	Product size (bp)	Reference
*fim*H	F: TGCTGCTGGGCTGGTCGATG R: GGGAGGGTGACGGTGACATC	52	550	[21]
*mrk*D	F: CCACCAACTATTCCCTCGAA R: ATGGAACCCACATCGACATT	47.5	226	[22]
*ent*B	F: ATTTCCTCAACTTCTGGGGC R: AGCATCGGTGGCGGTGGTCA	58.5	371	[20]
*wab*G	F: CGGACTGGCAGATCCATATC R: ACCATCGGCCATTTGATAGA	48.8	683	[20]
*uge*	F: GATCATCCGGTCTCCCTGTA R: TCTTCACGCCTTCCTTCACT	50.8	535	[20]
*irp*2	F: GCTACAATGGGACAGCAACGAC R: GCAGAGCGATACGGAAAATGC	56.7	230	[7]
*iuc*A	F: AATCAATGGCTATTCCCGCTG R: CGCTTCACTTCTTTCACTGACAGG	55	239	[7]
*peg*−344	F: CTTGAAACTATCCCTCCAGTC R: CCAGCGAAAGAATAACCCC	50	*508*	[7]
*Rmp*A	F: ACTGGGCTACCTCTGCTTCA R: CTTGCATGAGCCATCTTTCA	52	535	[23]
*Rmp*A2	F: CTTTATGTGCAATAAG-GATGTT R: CCTCCTGGAGAGTAAGCATT	48	451	[23]
*iro*B	F: CCCTGGCATATCAAAGGCGT R: GACAACAACGCGGGCATTTA	50	534	[24]
*bla*_OXA-48_	F: GCTTGATCGCCCTCGATT R: GATTTGCTCCGTGGCCGAAA	54	238	[25]
*bla*_NDM_	F: GGTTTGGCGATCTGGTTTTC R: CGGAATGGCTCATCACGATC	54	521	[25]
*bla*_KPC_	F: CATTCAAGGGCTTTCTTGCTGC R: ACGACGGCATAGTCATTTGC	54	498	[25]
*bla*_VIM_	F: GGTGTTTGGTCGCATATCGCAA R: ATTCAGCCAGATCGGCATCGGC	53	502	[26]
*bla*_IMP_	F: TCGTTTGAAGAAGTTAACGG R: ATGTAAGTTTCAAGAGTGATGC	46.5	568	[26]
K1	F: GTAGGTATTGCAAGCCATGC R: GCCCAGGTTAATGAATCCGT	50	1046	[23]
K2	F: GGAGCCATTTGAATTCGGTG R: TCCCTAGCACTGGCTTAAGT	53	1121	[23]
*mcr*−1	F: AGTCCGTTTGTTCTTGTGGC R: AGATCCTTGGTCTCGGCTTG	50	320	[27]

### Statistical analysis

The data was analyzed via Statistical Package for the Social Sciences version 26. For the qualitative data, numerical and percentage-based descriptive statistics were computed. To compare the different groups, we used the chi-square test (χ²-value) and Fisher’s exact test for the categorical data. A *p*-value < 0.05 was used to establish statistical significance. The R statistical platform (Version 64, 4.1.2, Toulouse, France) was used for creating the heatmaps.

### Nucleotide accession numbers

The sequenced genes were *bla*_KPC_, *bla*_NDM_, and *bla*_OXA-48_, added to GenBank with the accession numbers PQ356895, PQ356896, and PQ362692, respectively.

## Results

### Isolates characteristics

**Fig. 1:**
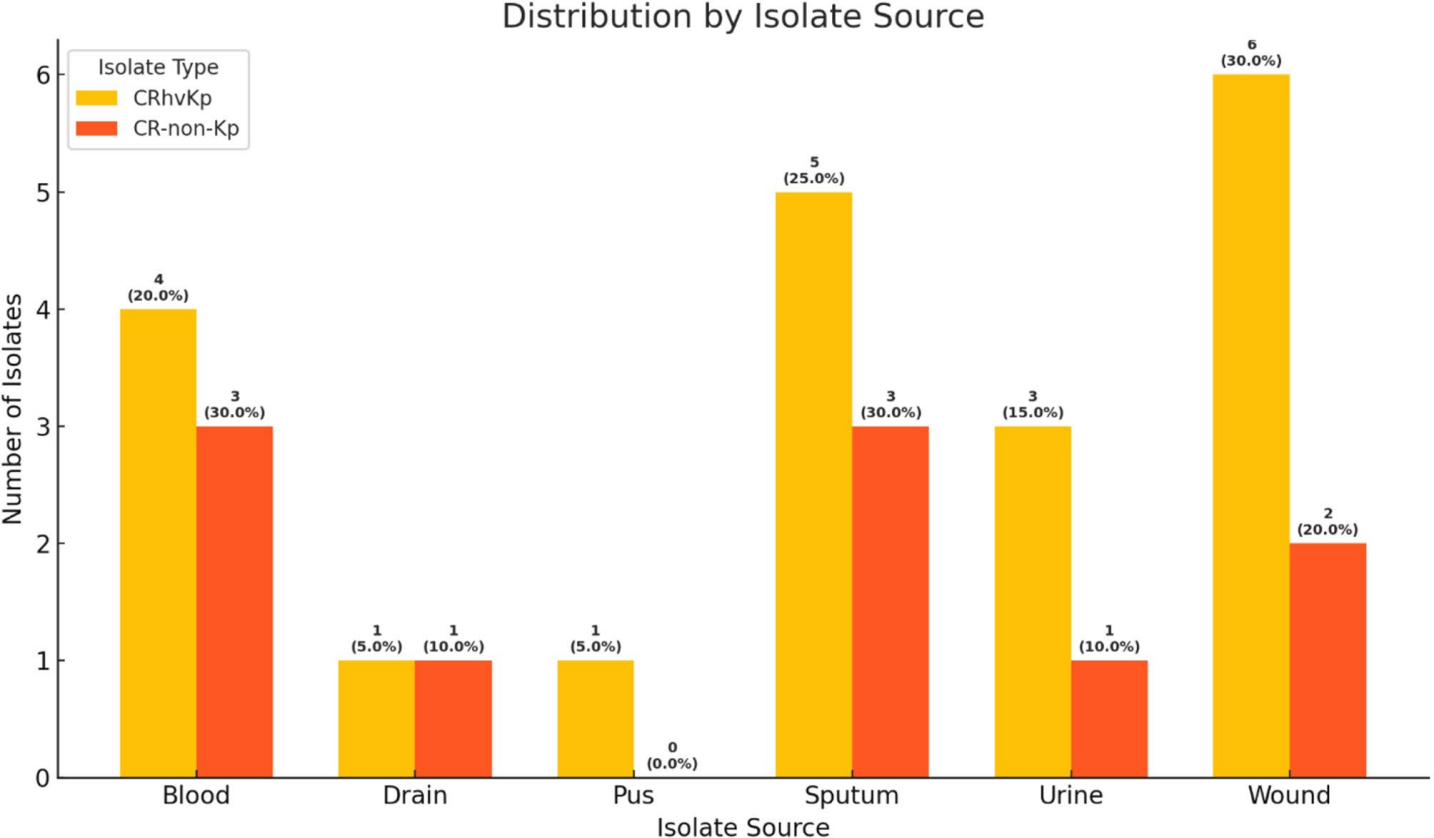
Distribution of CRKp isolates by isolate source

Out of 150 clinical isolates, 63 (42%) were confirmed by MALDI-TOF MS as *K. pneumoniae*, where 33/63 (52.4%) were collected from males and 30/63 (47.6%) from female hospitalized patients in Misr University for Science and Technology Teaching Hospital (Giza, Egypt). The *K. pneumoniae* isolates were obtained from pus 11/63 (17.5%), blood 14/63 (22.2%), sputum 13/63 (20,6%), urine 11/63 (17.5%), drain 2/63 (3.2%), and wounds 12/63 (19.1%). Of these, 30 isolates (30/63, 18.9%) were confirmed phenotypically and genotypically as CRKp isolates. The distribution of CRKp isolates by isolate source is shown in Please check if figures was captured and presented correctly.Fig. 1.

### Antimicrobial susceptibility testing

All 63 *K. pneumoniae* isolates exhibited a significant resistance pattern against a wide range of antimicrobial agents. Based on the CLSI 2023 breakpoints for carbapenem interpretation using disc diffusion zone diameters, 30 of the 63 *K. pneumoniae* isolates (47.6%) were resistant to meropenem and imipenem. Carbapenem resistance was identified in this study by resistance to at least one carbapenem agent. However, 90.5% (*n* = 57/63) of isolates remained susceptible to colistin. Resistance patterns to other antibiotics are summarized in Table 2. The antibiotic resistance patterns of CRhvKp and CR-non-hvKp isolates are represented in Table S1.

**Table 2:** Antibiotic resistance profile of isolates

Antibiotics	Resistant (*n*, %)	Intermediate (*n*, %)	Susceptible (*n*, %)
neomycin	60 (95.2%)	2 (3.2%)	1 (1.6%)
amoxicillin/clavulanate	59 (93.7%)	2 (3.2%)	2 (3.2%)
aztreonam	56 (88.9%)	2 (3.2%)	5 (7.9%)
nitrofurantoin	52 (82.5%)	3 (4.8%)	8 (12.7%)
piperacillin/tazobactam	52 (82.5%)	0 (0.0%)	11 (17.5%)
ceftazidime	50 (79.4%)	2 (3.2%)	11 (17.5%)
tetracycline	47 (74.6%)	7 (11.1%)	9 (14.3%)
trimethoprim-sulfamethoxazole	47 (74.6%)	2 (3.2%)	14 (22.2%)
ciprofloxacin	46 (73.0%)	11(17.5%)	6 (9.5%)
cefepime	45 (71.4%)	4 (6.3%)	14 (22.2%)
gentamycin	42 (66.7%)	3 (4.8%)	18 (28.6%)
azithromycin	38 (60.3%)	0 (0.0%)	25 (39.7%)
tobramycin	33 (52.4%)	2 (3.2%)	28 (44.4%)
imipenem	30 (47.6%)	4 (6.3%)	29 (46.0%)
meropenem	30 (47.6%)	2 (3.2%)	30 (47.6%)
amikacin	28 (44.4%)	2 (3.2%)	33 (52.4%)
colistin	6 (9.5%)	0 (0.0%)	57 (90.5%)

### Molecular characteristics of crkp isolates

**Fig. 2:**
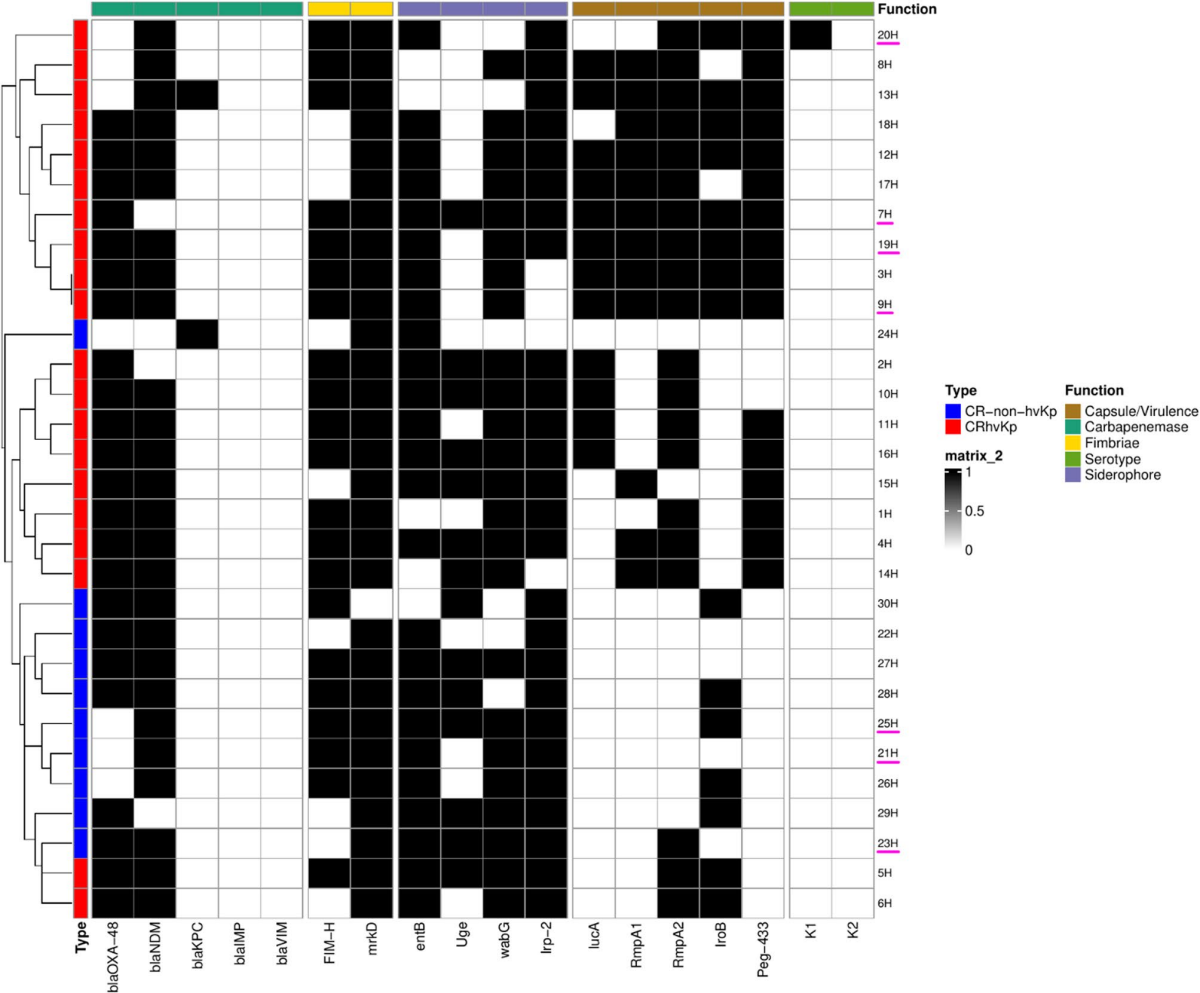
A Hierarchical clustering heatmap illustrating the prevalence of 19 resistance and virulence genes for 30 CRKp isolates is presented. The black box signifies the existence of genes, while the white box indicates their absence. Isolates are categorized by type: CRhvKp (red) and CR-non-hvKp (blue). Gene clusters are categorized by functional classification using color coding. (H) indicates the coding of the isolates. The X-axis clusters the clinical isolates, while the Y-axis groups genes with similar distribution patterns. Isolate codes underlined in pink indicate blood-source isolates

The prevalence of the five biomarker genes among our 30 CRKp isolates was as follows: *iuc*A (40%, 12/30), *rmp*A (40%, 12/30), *rmp*A2 (66.7%, 20/30), *peg*−344 (50%, 15/30), and *iro*B (50%, 15/30). We grouped these isolates into two categories, CRhvKp and CR-non-hvKp, based on whether they had *rmp*A or *rmp*A2 along with *peg*−344, *iro*B, and *iuc*A. The PCR test found that 20 out of 30 isolates (66.7%) were classified as CRhvKp and 10 out of 30 isolates (33.3%) as CR-non-hvKp (Fig. 2). Among the five biomarker genes, the predominant combination was *rm*pA + *rmp*A2 + *iro*B + *iuc*A + *peg*−344 (25%, 5/20), followed by *rmp*A + *rmp*A2 + *iuc*A + *peg*−344 (10%, 2/20), *rmp*A2 + *iro*B (10%, 2/20), *rmp*A2 + *iuc*A (10%, 2/20), *rmp*A + *rmp*A2 + *peg*−344 (10%, 2/20), *rmp*A2 + *iuc*A + *peg*−344 (10%, 2/20), *rmp*A + *iuc*A + *peg*−344 (5%, 1/20), *rmp*A + *rmp*A2 + *iro*B (5%, 1/20), *rmp*A2 + *iro*B + *peg*−344 (5%, 1/20), and *rmp*A2 *+ peg-*344 (5%, 1/20) (Fig. 2). Furthermore, positive outcomes were noted in the string test for 10% (2/20) of the CRhvKp strains and 10% (1/10) of the CR-non-hvKp strains(Figure S1).

The virulence genes were recognized among the CRhvKp and CR-non-hvKp strains: *fim*H (75%, 15/20 and 60%, 6/10), *mrk*D (100%, 20/20 and 90%, 9/10), *ent*B (80%, 16/20 and 90%, 9/10), *irp*2 (85%, 17/20 and 90%, 9/10), *wab*G (90%, 18/20 and 60%, 6/10), and *uge* (40%, 8/20 and 60%, 6/10). The distributions of these genes were not statistically significant in either the CRhvKp or CR-non-hvKp strains (*p* ≥ 0.05). For the capsular serotype genes (k1 and k2), we found just one positive k1 isolate in CRhvKp, and we did not observe any k2 serotypes in our isolates. The distribution of these genes was not statistically significant (Table 3).

**Table 3:** Molecular features of crkp isolates

Factors	CRKp (*n* = 30) n (%)	CRhvKp (*n* = 20) *n* (%)	CR-non-hvKp(*n* = 10) *n* (%)	*p*-value
Virulence genes				
* fim*H	22 (73.3%)	15 (75%)	6 (60%)	0.4
* mrk*D	29 (96.7%)	20 (100%)	9 (90%)	0.2
* ent*B	25 (83.3%)	16 (80%)	9 (90%)	0.5
* uge*	14 (46.7%)	8 (40%)	6 (60%)	0.3
* Wab*G	24 (80%)	18 (90%)	6 (60%)	0.06
* irp*2	26 (86.7%)	17 (85%)	9 (90%)	0.7
Stereotypes				
K1	1 (3.3)	1 (5%)	0 (0.0)	0.3
K2	0 (0.0)	0 (0.0)	0 (0.0)	---

Carbapenem Resistant *K. pneumoniae* (CRKp), Carbapenem Resistant Hypervirulent *K. pneumoniae* (CRhvKp), Carbapenem Resistant-non-Hypervirulent *K. pneumoniae *(CR-non-hvKp)

*Significant(*p*-values < 0.05)

### Phenotypic and genotypic distribution of carbapenemase genes in crkp

Based on phenotypic criteria (only mCIM positive), all CRKp isolates were carbapenemase producers (Table 4). The PCR results aligned with those of mCIM. All mCIM-positive isolates carried at least one carbapenem resistance gene. Carbapenemase genes found in CRhvKp included *bla*_OXA−48_ at 85% (*n* = 17/20) and *bla*_KPC_ at 5% (*n* = 1/20). The prevalence of these genes in CR-non-hvKp samples was as follows: *bla*_OXA−48_ at 60% (*n* = 6/10) and *bla*_KPC_ at 5% (*n* = 1/10). The gene frequencies showed no significant difference between the two groups (*P*-value > 0.05) (Table 4).

Approximately 47% of the isolates (*n* = 14/30) tested positive for both mCIM and eCIM. The phenotypic identification of MBL genes revealed findings of 40% (*n* = 8/20) for CRhvKp and 70% (*n* = 7/10) for CR-non-hvKp, as shown in (Table 4).

All samples that tested positive for mCIM/eCIM contained at least one MBL gene, according to the PCR results. In contrast to the phenotypic confirmatory criteria, a greater number of samples were found to be positive for MBL when using the PCR approach. Using PCR, 90% (*n* = 18/20) of CRhvKp and 80% (*n* = 8/10) of CR-non-hvKp strains tested positive for the *bla*_NDM_ gene. Conversely, both CRhvKp and CR-non-hvKp samples lacked the *bla*_VIM_ and *bla*_IMP_ genes. The occurrence of MBLs was not significantly different between the CRhvKp and CR-non-hvKp groups (*p-*value > 0.05, Table 4). The PCR results were validated using Sanger sequencing, with the following accession codes submitted to GenBank: KPC (PQ356895), NDM (PQ356896), and OXA (PQ362692).

**Table 4:** Phenotypic and genotypic recognition of carbapenemase genes among crkp, crhvkp, and CR-non-hvkp

Carbapenemase Detections	Total CRKp *n* = 30, n (%)	CRhvkp *n* = 20, n (%)	CR-non-hvkp *n* = 10, n (%)	*p*-value
Carbapenemase and MBLs				
Confirmation of phenotypic carbapenemase (only mCIM positive)	30 (100)	20 (100)	10 (100)	---
Genotypic carbapenemase confirmation via PCR	30 (100)	20 (100)	10 (100)	---
* bla*_OXA−48_	23 (76.7)	17 (85)	6 (60)	0.7
* bla*_kpc_	2 (6.7)	1(5)	1 (10)	0.5
Phenotypic MBLs confirmation (both mCIM/eCIM positive)	15 (50)	8 (40)	7 (70)	0.12
Genotypic MBLs confirmation using PCR	26 (86.7)	18 (90)	8 (80)	0.6
* bla*_NDM_	26 (86.7)	18 (90)	8 (80)	0.6
* bla*_IMP_	0 (0)	0 (0)	0 (0)	---
* bla*_VIM_	0 (0)	0 (0)	0 (0)	---

Carbapenem-resistant *K. pneumoniae* (CRKp), carbapenem-resistant hypervirulent *K. pneumoniae* (CRhvKp), carbapenem-resistant -non-hypervirulent *K. pneumoniae *(CR-non-hvKp)

*Significant *(p*-values < 0.05)

## Discussion

CRKp is currently considered a global health problem because of its significant role in mortality among patients with hospital-acquired infections. In this study, 30 isolates were selected based on their phenotypic and genotypic characteristics as CRKp isolates and were categorized into CRhvKp and CR-non-hvKp to investigate their molecular characteristics. The antimicrobial susceptibility assays were used to identify the CRKp isolates. All CRKp samples were MDR and showed strong resistance to common antibiotics like aztreonam, piperacillin-tazobactam, tobramycin, cefepime, amikacin, gentamicin, sulfamethoxazole, and cefoxitin, which supports results from an earlier study [11]. Colistin exhibits the lowest resistance rate, consistent with a prior report conducted in Iran [28].

Additionally, the rate of colistin resistance in our *K. pneumoniae* samples was 9.5% (6 out of 30), which is higher than what was found in an earlier study in Egypt that reported no colistin resistance in any samples [29]. Infections caused by carbapenem-resistant Enterobacteriaceae are ultimately treated with polymyxins, such as colistin and polymyxin B [30]. In 2015, the *mcr*−1 plasmid-mediated polymyxin resistance gene was initially discovered [31]. Our study showed no *mcr*−1 in any of our colistin-resistant isolates, in contrast to two studies conducted in Egypt that identified *mcr*−1 [32, 33]. The observed resistance to colistin may result from mechanisms other than the plasmid-mediated *mcr*−1 gene. Specifically, chromosomal mutations in the two-component regulatory systems PmrA/PmrB and PhoP/PhoQ, as well as alterations in the master transmembrane regulatory protein MgrB, have been implicated in colistin resistance [34, 35]. The modification of lipid A through the addition of phosphoethanolamine (pETN) and/or 4-amino-L-arabinose (L-Ara4N) increases its positive charges, thereby reducing its attraction for colistin [33]. Consequently, antibiotics must be used judiciously in the therapeutic management of CRKp infections to avert the emergence of novel drug resistance. To select the optimal antibiotic and limit the increase in antibiotic resistance, combination therapy must be prioritized, regardless of whether the CRKp infection is classified as hvKp or non-hvKp.

Our study revealed that among 30 CRKp isolates, the *bla*_NDM_ gene exhibited the highest prevalence of carbapenemase genes at 86.7%. This rate surpasses the documented statistics in Saudi Arabia (14%) [36] and Egypt (13%) [29]. Compared to prior studies in Saudi Arabia (82.2%) [36] and Egypt (17.39%), a lower proportion of isolates (63.6%) were positive for *bla*_OXA-48_ [29] In comparison to similar studies shown in Saudi Arabia [36] and Egypt (78.26%) [29].The prevalence of *bla*_KPC_ was found to be 6.7%, indicating an increase. Our findings align with the Egyptian study [37].

Reports suggest a rising incidence of CRhvKp cases over the past few years [23, 38, 39]. CRhvKp combines hypervirulence, multidrug resistance, and high transmissibility, posing significant health concerns to humans [8, 39, 40]. This study examined CRKp isolates for their antibiotic resistance profiles and molecular properties to elucidate the distinctions between CRhvKp and CR-non-hvKp.

The hypermucoviscous phenotype, linked to a positive string test, is a key feature of hvKp strains, with most CRhvKp testing positive [8, 41]. However, not all hvKp show this trait, while some classical K. pneumoniae do [42, 43], making the string test insufficient. More reliable hvKp markers include *peg*−344, *iuc*A, *iro*B, *rmp*A, and *rmp*A2 [7, 44]. In our study, only 2/20 CRhvKp and 1/10 CR-non-hvKp isolates were string-test positive (Figure S1), confirming the test’s low accuracy. A hypermucoviscous phenotype, mainly regulated by *rmp*A or *rmp*A2, was identified via the string test [11]. These genes control capsule synthesis [15]. One of the three positive isolates lacked *rmp*A/*rmp*A2, a phenomenon previously reported in clinical settings [38, 45]. To identify CRhvKp, we sought the virulence biomarker genes *rmp*A or *rmp*A2 alongside *iuc*A, *iro*B, or *peg*−344, consistent with a prior study [11].

As shown in Fig. 2, multiple combinations of virulence genes were identified among the isolates. Notably, either *rmp*A or *rmp*A2 was present in all five gene combinations, suggesting that these two genes may be essential virulence markers for defining CRhvKp. This observation is consistent with findings from a previous study [11]. Although these virulence gene combinations are effective in identifying hypervirulent *K. pneumoniae* (hvKp), the optimal combination of biomarkers for accurately predicting CRhvKp remains unclear.

This study investigated additional virulence genes in CRKp isolates and assessed their distribution between CRhvKp and CR-non-hvKp isolates. These virulence genes, including *wab*G, *mrk*D, *ent*B, *Irp*2, and *fim*H, were noticed in almost all CRKp isolates. This aligns with prior studies [46, 47], suggesting that these genes may serve as core pathogenic determinants in CRKp. Our results showed no significant difference in the prevalence of these virulence genes between CRhvKp and CR-non-hvKp groups. The capsule is widely recognized as a key virulence factor in *K. pneumoniae*, with a variety of K-antigen types contributing to its pathogenicity [48–50]. Serotypes K1 and K2 are particularly associated with hypervirulent strains and are the most reported and often linked to severe infections [51, 52]. Our investigation found no presence of the K2 gene in any isolate, and only one isolate exhibited the K1 gene, corroborating findings from a study conducted in China [53].

Our clustering analysis Fig. 2 indicated that *bla*_KPC_-positive isolates were distinct from most other CRKp strains, with reduced virulence gene content in at least one case. Specifically, two *bla*_KPC_-positive isolates (24 H and 13 H), illustrated in Fig. 2, showed contrasting virulence gene profiles. Isolate 13 H not only carried the common adhesin factors (fimH, mrkD) but also the siderophore marker entB and the five recognized hvKp biomarker genes (iucA, iroB, peg-344, rmpA, and rmpA2), which may collectively enhance its pathogenic potential. These genetic markers have been validated in several studies as reliable predictors of the hvKp phenotype, with strong associations to enhanced pathogenicity in both community and healthcare-associated infections [7, 15, 42].In contrast, isolate 24 H lacked these key hvKp determinants, resulting in a substantially reduced virulence gene repertoire despite harboring *bla*_KPC_. The coexistence of carbapenemase genes and hvKp markers is relatively uncommon but clinically significant, as it combines multidrug resistance with high virulence, increasing the risk of severe and difficult-to-treat infections [8, 11, 15, 36]. The observed divergence between our two *bla*_KPC_-positive isolates suggests that acquisition of carbapenem resistance in hvKp lineages may occur sporadically, potentially via horizontal gene transfer of resistance plasmids, and does not always result in a uniform virulence profile. This finding underscores the need for detailed molecular characterization including virulence gene profiling and plasmid analysis, to accurately distinguish *bla*_KPC_-producing strains with high epidemic potential from those with lower virulence.

Globally, the distribution of carbapenemase genes shows regional variation. In Europe, *bla*_KPC_ is among the most frequently detected carbapenemase genes in *K. pneumoniae* [13, 14], whereas in many African countries, including Egypt, its prevalence remains comparatively low, with *bla*_NDM_ and *bla*_OXA−48_ being the predominant carbapenemases [29, 52]. This pattern is consistent with surveillance data from the Middle East and North Africa, which report *bla*_NDM_ as the leading carbapenemase, followed by *bla*_OXA−48_, and a much lower occurrence of *bla*_KPC_ [28, 29, 52]. Our findings align with this epidemiological trend, showing a high prevalence of *bla*_NDM_ (86.7%), a moderate prevalence of *bla*_OXA−48_ (76.7%), and a low prevalence of *bla*_KPC_ (6.7%).

The following carbapenemase genes were identified in CRhvKp: *bla*_OXA−48_ (85%*)* and *bla*_NDM_ (90%); in CR-non-hvKp: *bla*_OXA−48_ (60%) and *bla*_NDM_ (80%). The prevalence of *bla*_OXA−48_ and *bla*_NDM_ in CRhvKp and CR-non-hvKp exceeds that reported in a study from Iran [28] which identified *bla*_NDM_ (25.0%, *n* = 2/8) and *bla*_OXA−48_ (0.0%) in CRhvKp. The prevalence of *bla*_NDM_ and *bla*_OXA−48_ among CR-non-hvKp was 41.3% and 20%, respectively. The *bla*_KPC_ gene was identified in 5% of isolates for both CRhvKp and CR-non-hvKp, which is significantly lower than the findings of a study from China (81.50% for CRhvKp and 69% for CR-non-hvKp) [11]. The distribution of carbapenemase genes exhibited no significant difference between CRhvKp and CR-non-hvKp isolates.

Analysis of the virulence and resistance gene heatmap revealed two distinct clusters of CR-non-hvKp and three lineages of CRhvKp. The CR-non-hvKp clusters were composed of isolates from diverse sources, including sputum, blood (3/10 isolates), wound, urine, and drain samples, with no clear temporal or ward-based clustering, suggesting sporadic distribution across the study period. In contrast, the CRhvKp lineages displayed more defined epidemiological patterns. One lineage was enriched in blood isolates (4/20), consistent with the known invasive potential of hvKp. A second lineage comprised mainly wound and pus isolates, most of which were recovered from surgical wards, while the third was dominated by respiratory (sputum) and drain samples, several originating from ICU patients. These findings suggest that while CR-non-hvKp strains appear widely distributed across sample types and time, although CRhvKp isolates were identified in ICU patients, this finding should not be interpreted as evidence of greater prevalence in hospital settings. More plausibly, it reflects the ability of CRhvKp infections to cause severe clinical deterioration necessitating ICU admission. Given the limited dataset, further studies are required to clarify this relationship.

Treatment protocols for carbapenem resistant hvKp are similar to those for carbapenem-resistant *K. pneumoniae*. However, while treating with tigecycline and polymyxin, one study found that the hypervirulent ST11-KL64 clone quickly evolved resistance [54]. In different research, it was proposed that ceftazidime-avibactam could be a viable option for treating hvKp isolates that are resistant to carbapenems, especially the ST11 hvKp isolates that produce KPC-2 [55]. But resistance to ceftazidime-avibactam has been developing in KPC-producing strains all over the world [56]. Alternative alternatives, including cefiderocol, should be evaluated. Cefiderocol, a novel siderophore cephalosporin developed to combat carbapenem-resistant bacteria, serves as an appropriate alternative for patients infected with carbapenemase-producing strains, including those that generate metallo-β-lactamases [57, 58]. The combination of ceftazidime-avibactam and aztreonam may be an appropriate alternative, especially in cases involving metallo-β-lactamase producers [59, 60]. These findings are consistent with the IDSA’s 2024 guidance on antimicrobial-resistant gram-negative infections [61].

In addition to appropriate antimicrobial therapy, infection control measures are critical to limit the spread of carbapenem-resistant *Klebsiella pneumoniae* (CRKp), particularly the hypervirulent strains. Standard precautions should be complemented by contact isolation, active surveillance cultures, and strict hand hygiene in healthcare settings [62]. Using antimicrobial stewardship programs (ASPs) also helps reduce the overuse of antibiotics and lowers resistance. Recent studies showed that combining these actions can greatly reduce CRKp infections in hospitals [63].

This study had several limitations. The samples were obtained from a single hospital, perhaps constraining the generalizability of the results. The relatively small sample size was due to the high cost associated with MALDI-TOF MS and VITEK^®^2 analysis. Furthermore, complete clinical data were unavailable for all instances due to restricted access to hospital records. To accurately identify the hvKp strain, it is essential to undertake both in vivo and in vitro experiments, utilizing models such as Galleria mellonella, murine models, and whole-genome sequencing. Future studies should include plasmid typing, including Inc-type characterization, which was not undertaken in the current study due to resource constraints.

## Conclusion

This study reports the emergence of MDR CRhvKp and CR-non-hvkp isolates holding different carbapenemases, including *bla*_OXA−48_, *bla*_KPC_, *bla*_NDM_, *bla*_IMP_, and *bla*_VIM_, in Egypt. CRhvKp, due to its hypervirulence and multidrug resistance, poses a significant threat to healthcare systems. CRhvKp has increasingly emerged as the predominant nosocomial pathogen. Hence, more effective treatment strategies and sustained monitoring are required to avoid the spread of CRhvKp and decrease selection pressure.

## Supplementary Information

Supplementary Material 1.

## Data Availability

The KPC, NDM, and OXA gene sequences\u0000have\u0000been submitted to NCBI GenBank under the accession numbers\u0000 PQ356895(https://www.ncbi.nlm.nih.gov/nuccore/PQ356895.1/), PQ356896(https://www.ncbi.nlm.nih.gov/nuccore/PQ356896), and PQ362692.(https://www.ncbi.nlm.nih.gov/nuccore/PQ362692.1/), respectively.

## Abbreviations

K. pneumoniae: Klebsiella pneumoniae
hvKp: hypervirulent K. pneumoniae
cKp: classical K. Pneumoniae
PLA: pyogenic liver abscesses
MDR: multidrug-resistant
rmpA: regulator of mucoid phenotype A
rmpA2: regulator of mucoid phenotype A2
iucA: Aerobactin
iroB: Salmochelin
peg-344: the putative metabolite transporter
irp2: yesrsinibactin
CRKp: carbapenem resistant K. pneumoniae
CRhvKp: carbapenem-resistant hypervirulent K. pneumoniae
CR-non-hvKp: carbapenem resistant-non-hypervirulent K. pneumoniae
entB: Enterobactin
mCIM: Modified carbapenem inactivation method
eCIM: EDTA Modified carbapenem inactivation method
MBLs: metallo-β-lactamases
CLSI: Clinical and Laboratory Standards Institute
PCR: Polymerase chain reaction
