# Supplementary material for: Coexistence of carbapenem resistance and hypervirulence in clinical Klebsiella pneumoniae isolates: a molecular and phenotypic analysis
Source: BMC Infect Dis. 2025 Sep 16;25:1109. doi: 10.1186/s12879-025-11585-z (PMC12439409; doi:10.1186/s12879-025-11585-z)
Supplement: Supplementary file 1 — Supplementary Material 1. [file 12879_2025_11585_MOESM1_ESM.docx]

**Coexistence of Carbapenem Resistance and Hypervirulence in Clinical *Klebsiella pneumoniae* Isolates: A Molecular and**

**Phenotypic Analysis**

Hadir Karam Ahmed ^1,2d^**^*^** , Fatma Molham^1b^  ,Ahmed O. El-Gendy^1c^, Mohamed Abd El-Gawad El-Sayed Ahmed^2a^

^1^Department of Microbiology and Immunology, Faculty of Pharmacy, Beni-Suef University, Beni-Suef 62514, Egypt

^2^Department of Microbiology and Immunology, Faculty of Pharmaceutical Sciences and Drug Manufacturing, Misr University for Science and Technology, Giza, 6^th^ of October City, Egypt

^a^ORCID:0000-0001-7428-8949, ^b^ORCID: 0000-0002-0980-5185,

^c^ORCID: 0000-0002-0151-2230, ^d^ORCID: 0000-0003-4753-7529.

Table S1 | Antibiotic resistance profile of **CR-hvKP and CR-non-hvKP strains against different antimicrobial agents**:

| Antibiotics | CRhvkp | | | CR-non-KP | | | p-value |
| --- | --- | --- | --- | --- | --- | --- | --- |
|  | R | I | S | R | I | S |  |
| SXT | 17 (85) | 0 (0) | 3 (15) | 7 (70) | 0 (0) | 3 (30) | 0.3 |
| AMC | 20 (100) | 0 (0) | 0 (0) | 10 (100) | 0 (0) | 0 (0) | --- |
| CN | 17 (85) | 2 (10) | 1 (5) | 8 (80) | 1 (10) | 1 (10) | 0.9 |
| CAZ | 19 (95) | 1 (5) | 0 (0) | 7 (70) | 0 (0) | 3 (30) | 0.03* |
| TPZ | 20 (100) | 0 (0) | 0 (0) | 7 (70) | 0 (0) | 3 (30) | 0.01* |
| AK | 18 (90) | 1 (5) | 1 (5) | 8 (80) | 0 (0) | 2 (20) | 0.4 |
| FEP | 18 (90) | 2 (10) | 0 (0) | 7 (70) | 1 (10) | 2 (20) | 0.1 |
| TE | 18 (90) | 2 (10) | 0 (0) | 8 (80) | 1 (10) | 1 (10) | 0.4 |
| IPM | 20 (100) | 0 (0) | 0 (0) | 10 (100) | 0 (0) | 0 (0) | --- |
| MEM | 20 (100) | 0 (0) | 0 (0) | 10 (100) | 0 (0) | 0 (0) | --- |
| F | 20 (100) | 0 (0) | 0 (0) | 10 (100) | 0 (0) | 0 (0) | --- |
| ATM | 20 (100) | 0 (0) | 0 (0) | 10 (100) | 0 (0) | 0 (0) | --- |
| CIP | 20 (100) | 0 (0) | 0 (0) | 10 (100) | 0 (0) | 0 (0) | --- |
| TOB | 20 (100) | 0 (0) | 0 (0) | 7 (70) | 0 (0) | 3 (30) | 0.01* |
| AZM | 18 (90) | 0 (0) | 2 (10) | 9 (90) | 0 (0) | 1 (10) | 1 |
| CT | 4 (20) | 0 (0) | 16 (80) | 2 (20) | 0 (0) | 8 (80) | 1 |

**R, resistant; I, intermediate; S, susceptible; CR-hvKP, carbapenem-resistant hypervirulent Klebsiella pneumoniae, carbapenem-resistant-non-hypervirulent *K. pneumoniae* (CR-non-hvKp).**

**AK, amikacin; AZM: Azithromycin; CAZ, ceftazidime; CIP, ciprofloxacin; CN, gentamycin; MER, meropenem; PTZ, piperacillin/tazobactam; TE, tetracycline; TOB, tobramycin; ATM, aztreonam; AMC, amoxicillin/clavulanate; CIP, ciprofloxacin; SXT, trimethoprim-sulfamethoxazole; TPZ, Piperacillin/tazobactam; IMI, imipenem; FEP, cefepime; F, Nitrofurantoin; N, Neomycin; CT, colistin.**

***Significant (P-values < 0.05)**

|  | Carbapenemases | | | | | Virulence genes | | | | | | Biomarkers | | | | | Capsular serotype | | String test |
| --- | --- | --- | --- | --- | --- | --- | --- | --- | --- | --- | --- | --- | --- | --- | --- | --- | --- | --- | --- |
| Gene | *bla*_OXA-48_ | *bla*_NDM_ | bla_KPC_ | *bla*_IMP_ | *bla*_VIM_ | *FIM*-H | *mrk*D | *ent*B | *Uge* | WabG | *Irp*_2 | *Iuc*A | *Rmp*A1 | *Rmp*A2 | *IroB* | *Peg*-433 | K1 | K2 |  |
| 1H |  |  |  |  |  |  |  |  |  |  |  |  |  |  |  |  |  |  |  |
| 2H |  |  |  |  |  |  |  |  |  |  |  |  |  |  |  |  |  |  |  |
| 3H |  |  |  |  |  |  |  |  |  |  |  |  |  |  |  |  |  |  |  |
| 4H |  |  |  |  |  |  |  |  |  |  |  |  |  |  |  |  |  |  |  |
| 5H |  |  |  |  |  |  |  |  |  |  |  |  |  |  |  |  |  |  |  |
| 6H |  |  |  |  |  |  |  |  |  |  |  |  |  |  |  |  |  |  |  |
| 7H |  |  |  |  |  |  |  |  |  |  |  |  |  |  |  |  |  |  |  |
| 8H |  |  |  |  |  |  |  |  |  |  |  |  |  |  |  |  |  |  |  |
| 9H |  |  |  |  |  |  |  |  |  |  |  |  |  |  |  |  |  |  |  |
| 10H |  |  |  |  |  |  |  |  |  |  |  |  |  |  |  |  |  |  |  |
| 11H |  |  |  |  |  |  |  |  |  |  |  |  |  |  |  |  |  |  |  |
| 12H |  |  |  |  |  |  |  |  |  |  |  |  |  |  |  |  |  |  |  |
| 13H |  |  |  |  |  |  |  |  |  |  |  |  |  |  |  |  |  |  |  |
| 14H |  |  |  |  |  |  |  |  |  |  |  |  |  |  |  |  |  |  |  |
| 15H |  |  |  |  |  |  |  |  |  |  |  |  |  |  |  |  |  |  |  |
| 16H |  |  |  |  |  |  |  |  |  |  |  |  |  |  |  |  |  |  |  |
| 17H |  |  |  |  |  |  |  |  |  |  |  |  |  |  |  |  |  |  |  |
| 18H |  |  |  |  |  |  |  |  |  |  |  |  |  |  |  |  |  |  |  |
| 19H |  |  |  |  |  |  |  |  |  |  |  |  |  |  |  |  |  |  |  |
| 20H |  |  |  |  |  |  |  |  |  |  |  |  |  |  |  |  |  |  |  |
| 21H |  |  |  |  |  |  |  |  |  |  |  |  |  |  |  |  |  |  |  |
| 22H |  |  |  |  |  |  |  |  |  |  |  |  |  |  |  |  |  |  |  |
| 23H |  |  |  |  |  |  |  |  |  |  |  |  |  |  |  |  |  |  |  |
| 24H |  |  |  |  |  |  |  |  |  |  |  |  |  |  |  |  |  |  |  |
| 25H |  |  |  |  |  |  |  |  |  |  |  |  |  |  |  |  |  |  |  |
| 26H |  |  |  |  |  |  |  |  |  |  |  |  |  |  |  |  |  |  |  |
| 27H |  |  |  |  |  |  |  |  |  |  |  |  |  |  |  |  |  |  |  |
| 28H |  |  |  |  |  |  |  |  |  |  |  |  |  |  |  |  |  |  |  |
| 29H |  |  |  |  |  |  |  |  |  |  |  |  |  |  |  |  |  |  |  |
| 30H |  |  |  |  |  |  |  |  |  |  |  |  |  |  |  |  |  |  |  |

**Figure S1: string test, biomarkers, virulence genes, capsular stereotype, and carbapenemase genes among CRKp isolates. Gene presence in carbapenem resistant hypervirulent *K. pneumoniae* (CRhvKp) is indicated by blue boxes, while gene presence in carbapenem resistant -non-hypervirulent *K. pneumoniae* (CR-non-hvKp) is shown in green boxes. Light gray boxes denote gene absence.**
